# Beating the Bio-Terror Threat with Rapid Antimicrobial Susceptibility Testing

**DOI:** 10.3390/microorganisms9071535

**Published:** 2021-07-19

**Authors:** Shahar Rotem, Ida Steinberger-Levy, Ofir Israeli, Eran Zahavy, Ronit Aloni-Grinstein

**Affiliations:** Department of Biochemistry and Molecular Genetics, Israel Institute for Biological Research, Ness-Ziona 74100, Israel; shaharr@iibr.gov.il (S.R.); idasl@iibr.gov.il (I.S.-L.); ofiri@iibr.gov.il (O.I.); eranz@iibr.gov.il (E.Z.)

**Keywords:** bioterror, *Bacillus anthracis*, *Yersinia pestis*, *Francisella tularensis*, antibiotic, clinical samples, environmental samples, blood cultures, genotypic tests, phenotypic tests, high throughput sequencing

## Abstract

A bioterror event using an infectious bacterium may lead to catastrophic outcomes involving morbidity and mortality as well as social and psychological stress. Moreover, a bioterror event using an antibiotic resistance engineered bacterial agent may raise additional concerns. Thus, preparedness is essential to preclude and control the dissemination of the bacterial agent as well as to appropriately and promptly treat potentially exposed individuals or patients. Rates of morbidity, death, and social anxiety can be drastically reduced if the rapid delivery of antimicrobial agents for post-exposure prophylaxis and treatment is initiated as soon as possible. Availability of rapid antibiotic susceptibility tests that may provide key recommendations to targeted antibiotic treatment is mandatory, yet, such tests are only at the development stage. In this review, we describe the recently published rapid antibiotic susceptibility tests implemented on bioterror bacterial agents and discuss their assimilation in clinical and environmental samples.

## 1. Introduction

The ongoing COVID-19 pandemic is causing a staggering loss of life and health, together with an economic collapse, anxiety, and social disorder. Indeed, the entire world has become aware of the impact of a pandemic on our daily life [1]. The worldwide battle to block the COVID-19 pandemic has uncovered the susceptibility of global societies to natural pathogens as well as to intentionally released bioterror agents. Moreover, the COVID-19 pandemic may have a long-term secondary impact on medical issues such as antibiotic resistance [2]. By highlighting the threats and costs of a pandemic, COVID-19 has raised queries about our preparedness for a bioterror attack. Now, more than ever is the time to develop strategies and means for bioterror preparedness.

Bioterror is not a new threat. It is believed that already in the 14th century BC the Hittites used bioterror by sending *Francisella tularensis* infected rams to their enemies [3]. Similarly, snake venom, *Clostridium perfringens*, *Clostridium tetani*, infected individuals or arrows covered with *Yersinia pestis*, distribution of blankets from a smallpox infected hospital, and the use of saliva from rabid dogs are all historical examples of biological warfare [4].

Currently, the Centers for Disease Control and Prevention (CDC) categorizes potential biological agents into three categories, based on their severity. High-priority agents (Tier-1, category A) include pathogens that pose a risk to national security as they can be easily disseminated or transmitted from person to person, result in high mortality rates, have the potential for major public health impact, might cause public panic and social disruption, and require special action for public health preparedness. Among category A agents are viruses, such as Variola major (smallpox), Filoviruses (Ebola, Marburg), Arenaviruses (Lassa, Machupo), Bunyaviruses (Congo-Crimean, Rift Valley) and Flaviviruses (Dengue), and bacteria, including *Bacillus anthracis* (anthrax), *Y. pestis* (plague) and *F. tularensis* (tularemia) and the *Clostridium botulinum* toxin [5].

Identification of the agent is the first step towards proper treatment and mitigation of a bioterror event. In the case of a bacterial pathogen, antibiotics will be offered to the infected patients. As agents used by bioterrorists may be genetically engineered to resist current therapeutic antibiotics [6], an antibiotic susceptibility test (AST) should assure that clinically relevant antibiotics are prescribed both to infected patients as well as a prophylactic treatment to the potentially exposed public. Herein, we discuss the efforts and achievements made to develop rapid ASTs for bioterror agents, focusing on Tier-1 bacteria, *B. anthracis*, *Y. pestis*, and *F. tularensis.* We will discuss ASTs that are suited for clinical samples and emphasize the benefits of ASTs for environmental samples, which may offer rational targeted prophylactic treatment, even before the onset of morbidity in exposed individuals, thus allowing mitigation of the event with minimum casualties.

## 2. Bacterial Bioterror Agents—*B. anthracis*, *Y. pestis*, and *F. tularensis*

*B. anthracis* is the causative agent of anthrax, a highly contagious and deadly disease that can infect humans by respiratory, cutaneous, or gastrointestinal routes [7]. Respiratory anthrax is the most severe form of the disease and without adequate antibiotic treatment mortality rates are over 90%. However, prompt diagnosis and rapid and adequate antibiotic treatment will significantly lower those rates [8]. The CDC recommends prophylaxis with ciprofloxacin (500 mg PO bid) or doxycycline (100 mg PO bid) after inhalational exposure [9]. Yearly, between 2000 and 20,000 human anthrax patients are reported in various locations in the world including India, Pakistan, Bangladesh, Zimbabwe, United States, South Africa, Iran, Turkey, and more [10]. *B. anthracis* survives in the soil in the form of spores, is highly resilient to environmental insults, and therefore is able to survive for eras. Spores, which represent the infectious form of the bacteria, can potentially be maliciously used as a bio-threat weapon [10].

*Y. pestis* represents a serious problem for worldwide public health either through the small outbreaks of plague that occur throughout the world [11,12,13] or by its potential use, via the aerosol route of exposure, as a bioterrorism agent that might cause mass casualties [14]. An estimation by the World Health Organization (WHO) suggests that if 50 kg of the plague pathogen were to be released as an aerosol over a city with a population of 5 million, 150,000 people might fall ill with pneumonic plague, 36,000 of whom would die [15]. High mortality rates occur if prompt treatment is not initiated early following exposure [14]. No safe and efficient vaccine is available and treatment relies on antibiotics. The recommended antibiotic treatment for plague includes streptomycin, tetracycline, and chloramphenicol [15]. Other antibiotics that can be used to treat plague are gentamicin, levofloxacin, ciprofloxacin, and moxifloxacin, [16]. For a recent comprehensive review on antibiotic therapy of plague please see [17].

Clinical isolates of plasmid-mediated single and multiple drug-resistant strains of *Y. pestis* have been reported [18,19] as well as non-virulent, spontaneous mutant isolates with reduced susceptibility to ciprofloxacin [20,21], moxifloxacin [22], levofloxacin [23], streptomycin [23], and doxycycline were selected in vitro [24]. Thus, AST should be carried out to verify the correct antibiotic treatment.

*F. tularensis* is a highly infectious organism. Inhalation of as few as 10 colony-forming units is sufficient to cause disease in humans with fatality rates of up to 30% to 60% if improperly treated. As a bioterror agent, the bacterium could be disseminated by aerosols leading to a high number of severe pneumonia cases and the contamination of water and soil for prolonged periods, which may cause secondary infections in human beings and animals [25,26]. A deliberate release of aerosolized *F. tularensis* over London was estimated to result in 2.4 million exposures, 130,000 infections, and 24,000 deaths [27]. To date, there is no safe and efficient vaccine for tularemia, thus, treatment relies on antibiotics. Natural resistance is seen for β-lactams and macrolides for type B biovar II strains [26]. First-line treatment includes aminoglycosides such as streptomycin and gentamicin, tetracyclines, for instance, doxycycline, and fluoroquinolones like ciprofloxacin and levofloxacin [28]. Unfortunately, the overuse of fluoroquinolones in the last two decades has led to treatment failure and relapses in tularemia patients [29]. Moreover, as *F. tularensis* is a facultative intracellular bacterium, any offered antibiotic should have intracellular pharmacokinetic and pharmacodynamic activity [30,31]. To note, resistant strains can be easily selected in vitro [32,33,34,35,36], thus ASTs should be applied prior to treatment selection, especially following a bioterror event.

### 2.1. Traditional ASTs

Currently, the gold standard for determining antimicrobial susceptibility for biothreat agents is the conventional broth microdilution (BMD) method which is based on the Clinical and Laboratory Standards Institute guidelines [37]. This method is growth- dependent and requires an incubation period of 16 to 20 h for *B. anthracis*, 24 to 48 h for *Y. pestis*, and 48 to 72 h for *F. tularensis* [37]. Other methods commonly used for AST are agar dilution and diffusion-based assays such as the disc diffusion and the Etest. These alternative methods, although easier to handle/practice, require similar incubation times since visible growth is required for the interpretation of the results.

Reports of antimicrobial susceptibility profiles of *B. anthracis* are scarce. This is probably due to the rarity of human anthrax cases in developed countries. A comparison of standard broth microdilution and Etest agar diffusion on 65 *B. anthracis* isolates, conducted by the CDC [38], found that there was no statistically significant difference between both methods for any of the antimicrobial agents tested, except for penicillin in which the Etest method was between 1 to 9 two-fold dilutions lower than the standard broth microdilution method.

*Y. pestis* is a fastidious bacterium, thus it has a low growth rate on artificial media, and so susceptibility testing methods for *Y. pestis* have been difficult to standardize. In a study that compared two different AST methods, for *Y. pestis* susceptibility testing [38], it was found that for ciprofloxacin, doxycycline, gentamicin, levofloxacin, streptomycin, and tetracycline, the Etest and BMD correlated well except for chloramphenicol and trimethoprim-sulfamethoxazole, for which endpoint readings were difficult to determine by Etest. They also recommended that nonsusceptible Etest MICs be confirmed by a reference BMD test.

There have been several reports of *F. tularensis* ASTs from reference laboratories [39,40,41,42,43,44,45,46,47,48,49,50,51,52]. However, the AST procedures reported in the literature were poorly standardized between studies and AST data have not been previously evaluated in an inclusive and comparative way. A review, which collected *F. tularensis* AST results from several studies containing data from 898 *F. tularensis* strains isolated from humans, animals, arthropods, natural water samples, and unknown sources, aimed to summarize and standardize the data [53]. The authors concluded that it is crucial to follow CLSI guidelines and that the broth microdilution technique using enriched cation-adjusted Muelle–Hinton broth (caMHB) medium should be considered as the reference method. The modified Mueller–Hinton II liquid medium should not be used for *F. tularensis* AST to avoid reporting MICs that could categorize strains as resistant to aminoglycosides or doxycycline. They also recommended that reference laboratories should use the broth microdilution method to test large collections of *F. tularensis* strains, while the Etest can be used for testing one or a few strains, however, still requiring comparative studies with the reference method.

### 2.2. Isolation Procedures

Efficient and selective plating media is an integral part of the bacterial isolation process and is also one of the major requirements of the CLSI in order to achieve an appropriate MIC value of the tested antibiotic toward the targeted bacteria. However, this is also one of the major bottlenecks, which largely elongates the time needed for antibiotic susceptibility determination. Finding new techniques to shorten this stage will be a significant step toward a shorter test. There are several works that targeted this issue in various ways which include improved selective and rapid culturing or immuno-physical rapid separation methods via magnetic beads or Fluorescent Activated Cell Sort (FACS). It should be mentioned that the European Committee on Antimicrobial Susceptibility Testing (EUCAST) has recently issued guidelines for rapid antimicrobial susceptibility testing (RAST) performed directly from positive blood culture vials [54]. We recently showed that this direct assay can be applied on the three Tier-1 agents [55].

#### 2.2.1. Selective Culturing

##### *B.* *anthracis*

The initiative to develop a selective media for *B. anthracis* started over 50 years ago. A paper from 1951 reports that 4: 4-diamidino-diphenoxypropane (propamidine) allowed the germination of *B. anthracis* spores whereas it repressed the growth of practically all other microorganisms tested [56]. Another paper describes an agar plate medium, named PLET, which contains the following ingredients added to Difco Heart Infusion Agar (HIA): polymyxin, lysozyme, disodium ethylenediaminetetraacetate, (EDTA), and thallous [57]. These plates seem to be superior to other plates for the isolation of *B. anthracis* from other spore-forming bacilli [58]. There are some limitations for this medium such as stricter safety and environmental regulations due to the presence of thallium acetate [59], the ability of other *Bacillus* strains to grow on the PLET agar [60], and the reduction in the germination of *B. anthracis* spores on this media [61]. Recently, a novel selective media for *B. anthracis*, named CEFOMA (Bacillus CEreus sensu lato group-specific antibiotics, FOsfomycin, MAcrolides), was developed [62]. This media enabled the growth of all tested *B. anthracis* strains while inhibiting all other species within the *B. cereus* sensu lato group, facilitating the isolation of *B. anthracis* from spiked soil samples.

##### *Yersinia* *pestis*

Attempts to develop a selective media for *Y. pestis* were initiated already early in the 20th century [63,64,65]. Of the WHO-recommended media for the isolation of *Y. pestis,* MacConkey agar holds the best selective traits mainly due to ingredients such as crystal violet, which inhibits some gram-positive organisms, as well as bile salts that inhibit the growth of non-enteric bacteria. However, slow growth of *Y. pestis* is observed as a consequence of these selectivity agents [66].

Cefsulodin-irgasan-novobiocin (CIN) agar [67], is another media used for the isolation of *Y. pestis*. As with MacConkey, only some of the platted bacteria grow to form colonies [68]. Thus, the tradeoff of growth support versus selectivity is a major concern in these selective media. To improve the balance between growth supports to selectivity we developed a novel improved medium named BIN that has high selectivity, yet holds growth support for *Y. pestis* [66]. An improved version of the BIN medium was recently published [69]. The preparation of the new medium is less complex and its performance was found to be superior to that of first-generation BIN medium. In Madagascar, approximately 20% more *Y. pestis* positive isolates were identified by the improved-BIN medium compared to the commercially CIN selective medium [69].

##### *Francisella* *tularensis*

*F. tularensis* is a slow-growing bacterium, thus its isolation from other fast-growing contaminating bacteria is mandatory in order to obtain a correct MIC value. The use of selective media is an option. It was shown that the isolation of *F. tularensis* from throat swabs, a wound in the hand, or from lymph node aspirate was achieved by the addition of antibiotics such as penicillin, polymixin B sulfate, and cycloheximide to cysteine heart agar (CHA) with 2.5% human blood suppressing the growth of normal flora [70]. CHA with chocolatized 9% sheep blood (CHAB), supplemented with 7.5 mg of colistin, 2.5 mg of amphotericin, 0.5 mg of lincomycin, 4 mg of trimethoprim, and 10 mg of ampicillin per liter (CHAB-A) was also shown to enhance recovery of *F. tularensis* from tissue samples [71]. A combination of antimicrobials with broad range activity against either Gram-negative or Gram-positive bacteria or fungi, but not *Francisella* spp. (polymyxin B, amphotericin B, cefepime, cyclohexamide, and vancomycin) was used to recover *F. tularensis* from environmental samples [72]. Others have suggested the use of acid treatment and a selective medium to enhance the recovery of *F. tularensis* from water [73]. Indeed, these procedures reduced contaminating bacteria, yet, they are time-consuming and require 2–3 days of incubation on an agar plate. Procedures to isolate the bacteria within shorter periods will be beneficial.

#### 2.2.2. Rapid Bacterial Isolation: Plasma Purification and Immunomagnetic Separation

Conducting AST for bacteria grown in blood culture, requires its isolation from blood cells and plasma components, as they interfere with the absorbance measurement. Thus, in a standard AST, bacteria are purified and enriched on agar plates. Shortening this step was shown for both *F. tularensis* and *Y. pestis* by using the serum separation tube (SST) [74,75]. *F. tularensis* is predominantly present in the extracellular fraction of blood culture samples, thus its isolation can be simply reached by using serum separation tubes [74,75]. Following, an application of an immunomagnetic separation (IMS) procedure, using *F. tularensis* or *Y. pestis* specific antibodies, allowed the separation of the bacteria from the blood components and growth-inhibitory substances present in the serum fraction. Isolated bacteria were transferred into proper enrichment media, which permitted bacterial growth to the desired bacterial load for the chosen AST. The IMS procedure was found to be efficient, ranging in most cases between 90–100% [74]. Within only 1 h, *Y. pestis* and *F. tularensis* were isolated for further AST procedures as an alternative to the 1–2 days required for agar platting, respectively. This reduction of time is reflected in the overall time of the AST.

#### 2.2.3. Rapid Bacterial Isolation by Fluorescent Activated Cell Sorter (FACS)

The use of Fluorescent Activated Cell Sorter (FACS) for the isolation of targeted pathogenic bacteria from environmental or clinical samples has always been challenging mainly due to limitation in the optical capabilities of the FACS to observe small bacteria in the complex noise of the samples, the pretreatment step of the samples, and of course the use of the correct antibodies and fluorophore to achieve selective isolation. We reported on the development of a FACS procedure for the isolation of *B. anthracis* spores from environmental samples [76,77] where it was shown that by using selective antibodies for double labeling or FRET labeling of the spores, one can achieve a highly selective gate for the spores and gain a rapid collection of ca 1 × 10^6^ spores per 10 min out of samples with spore concentration of ca 10^6^ spores/mL with contaminants of up to 10^5^ cfu/mL. The full process was further developed to be robust and included pretreatment of the samples by immunomagnetic separation of the spores from samples (Section 2.2.2). The entire procedure consisted of an IMS step, followed by an immune fluorescent labeling and a final heat-shock step. The process yielded, within less than 10 h, an isolated spore sample ready for AST analysis. The same methodology was implemented successfully to *Y. pestis* samples [78] allowing isolation within significantly shorter time frames.

### 2.3. New Rapid ASTs

**Figure 1:**
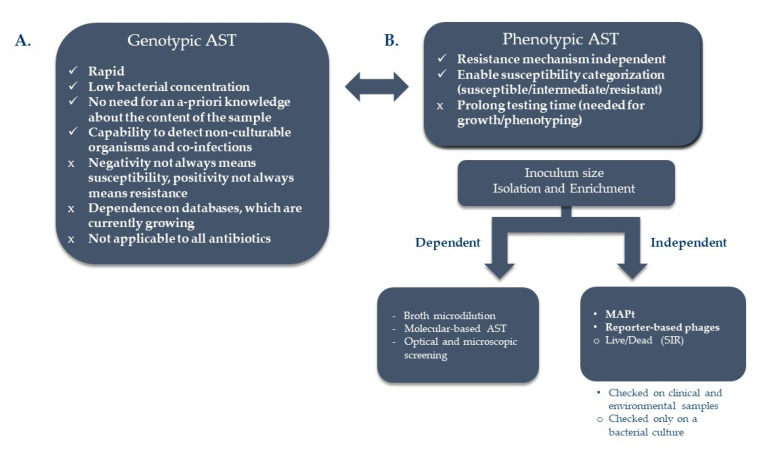
Advantages and disadvantages of the various ASTs. ASTs may be based on genotypic (**A**) or phenotypic measurements (**B**). The main limitations of many of the phenotypic ASTs are the dependence of inoculum size and the time-consuming isolation/enrichment steps. MAPt and reporter-based phage ASTs are promising candidates for ASTs that overcome these limitations.

Shorter isolation steps are beneficial timewise, yet, the standard ASTs that follow have their limitations, requiring in all, a day to a few days, depending on the growth rate of the tested bacteria. In the past years, attempts were made to develop new and rapid ASTs, some of which were implicated on the three bio-terror agents: *B. anthracis*, *Y. pestis,* and *F. tularensis* while some were initially developed for these bio-terror pathogens. These include genotypic-based high throughput sequencing and emerging databases that use newly developed algorithms to identify antibiotic resistance signatures and phenotypic- based detection of early response alterations within the bacteria or growth, following antibiotic exposure, using rapid means (Figure 1).

#### 2.3.1. Genotypic-Based Assays—High Throughput Sequencing

Genetic identification of bio-threat agents without a priori knowledge is a challenging task, moreover, determining its antibiotic susceptibility profile. In the relatively simple scenario where there is a solid suspicion of a specific pathogen and its susceptibility profile is well known, PCR is the method of choice in most cases, allowing the amplification of specific targets. These methods allow a rapid, straightforward, and highly sensitive identification within minutes to a few hours, from only minuscule quantities of the target genome in the sample. Nevertheless, PCR-based techniques require some level of previous data, which is not always present. In the event where no prior information about the content of a sample and its susceptibility profile can be found or when there is a suspicion of an emerging or genetically engineered pathogen outburst, the definitive nucleic acid-based detection method should be based on unprejudiced DNA sequencing. Sequencing of the complete content of a sample can deliver not just an answer for the ‘yes or no question’ of whether an explicit pathogen is present in the sample but likewise reply the ‘what question’, the query of the sample content, together with the description of diverse genomic traits, such as its susceptibility profile [79,80,81]. Until the previous decade, utilizing DNA sequencing to interpret the content of an unknown sample and its traits was not possible. Sanger-based sequencing methods were not suitable for studying mixtures and were high-cost, as well as time and labor-consuming. The development, a decade ago, of high-throughput sequencing (HTS; also termed 2nd generation sequencing) techniques, paved the way for complete detection of pathogens without any preceding information. These immense parallel sequencing platforms can sequence a varied mixture of genetic ingredients with ultra-high sensitivity and rapidity and with a lesser price per base equated with the previous techniques [82,83]. We showed the ability to identify *Y. pestis* and *B. anthracis* spike-ins, which served as simulants for unknown pathogens, in whole blood and environmental samples, using HTS at relevant bacterial concentrations (down to 10^3^/mL) and within a timeframe of a working day. This result was accomplished by a swift library preparation process, short-length sequencing, and a rapid bioinformatics comparison against all existing microbial genomic sequences [84,85].

The development of 3rd generation long read-based sequencing platforms (such as Oxford nanopore and Pacific Biosciences) which produce reads in the ten’s kb range is currently accelerating the field even further. These methods can shorten the sequencing process to a few minutes, analyze also repetitive regions, and permit complete bacterial genome sequence finishing. Nevertheless, long-read sequence data have a significantly higher error rate than the 2nd generation HTS platforms and require a high amount (in the micrograms range) of nucleic acids for genomic library preparation.

HTS has other advantages apart from the enhanced discovery of known and unknown bio-agents in diverse samples; amid these are the capability to detect non-culturable microorganisms and the capacity to detect co-infections, drug resistance, and antibiotic resistance. Moreover, HTS is the only current genetic-based method that could detect genetically modified or engineered organisms [86,87]. Although there is not a complete knowledge of all genes or mutations associated with antibiotic resistance, HTS is invaluable for understanding novel genotypic/phenotypic relationships. Vast efforts are currently ongoing to combine the advancements in HTS, detailed above, alongside phenotypic antimicrobial susceptibility data to create comprehensive genotype–phenotype databases for the prediction of antibiotic susceptibility motifs. Those efforts, reviewed in [88,89,90], yielded more than 50! databases that use newly developed algorithms to analyze genomic information to predict phenotypes and facilitate the understanding of the connections between DNA variation and antibiotic susceptibility. The databases include inter alia ResFinder (now in version 4.0): the initial online bioinformatics tool intended for users lacking expert bioinformatics skills, which detects antibiotic susceptibility genes in HTS data that is submitted via a web server [91]; VampR: variant mapping and prediction of antibiotic resistance through understandable features and machine learning [92]; ARIBA: rapid antimicrobial resistance genotyping straight from sequencing reads [93]; AMRFinder from NCBI [94]; and MEGARes 2.0: a database for classification of the antimicrobial drug, biocide and metal resistance determinants in metagenomic sequence data [95]. In these databases, novel resistance elements from genomic data are being pursued using different approaches combining machine learning and artificial intelligence strategies. The ultimate goal of such strategies is the correct and rapid prediction of susceptibility not only in known pathogens or bioterror agents but also as a prediction of antibiotic resistance motifs in unknown, emerging, and genetically engineered agents. This goal, which seemed to be beyond scope a few years ago, could be conducted currently in a working day [84,85,96].

#### 2.3.2. Phenotypic Based ASTs

Genotypic assays can detect the presence of resistance elements but not determine susceptibility. At present, rather than substituting phenotypic susceptibility testing, genotypic testing can supplement it. Moreover, in several cases, the absence of a resistance gene does not essentially predict susceptibility to a specific drug and the existence of a resistant gene does not assure resistance, as in the case of *B. anthracis*, which has two β-lactamase genes on the chromosome. These genes are not expressed in most strains, and the organisms remain susceptible to β-lactam antibiotics [97]. Thus, functional phenotypic susceptibility testing is still required.

##### 2.3.2.1. Live/Dead Fluorescent Detection

Fluorescent probes for live/dead detection of bacterial viability are in vast use in many bacterial analyses using fluorescent microscopy or flow cytometry. For example, Nuding et al. reported on a rapid fluorescence AST method that monitored bacterial viability by determining membrane potential using the oxonol dye, DiBAC4, as a fluorophore indicator [98]. Though it was shown that each antibiotic agent primes a different inactivation path, using a membrane potential probe permits the forecast of bacterial viability. Nevertheless, this technique necessitates prolonged incubation of the dye with the bacteria and cumbersome washing steps. Moreover, a reliable real-time prediction of the bacterial viability state by fluorescent labels, under diverse conditions that cause cell death, among them antibiotics, involves a more complex blend of fluorophores; thus, a single dye is not sufficient for all inactivation methods [99,100]. Moreover, since various antibiotic agents activate different antibacterial mechanisms, for instance, cell wall damage, protein synthesis inhibition, or DNA destruction, leading to massive bacterial response mechanisms, the use of a single dye detector is not trivial. We have recently adopted a new fluorescent labeling method, named SIR (Spectral Intensity Ratio) using an oxonol dye (synapto green/FM1-43) that was shown to be efficient in identifying Gram-negative bacterial death triggered by a variety of death methods for [101]. This new finding led to the development of both a new and rapid AST in the clinical field [102] and for the pathogenic bacteria *Y. pestis* [103]. By using FM1-43 staining of 5 × 10^4^ to 5 × 10^6^ cfu/mL *Y. pestis* bacteria, exposed to a series of different concentrations of various antibiotics, we were able to determine the MIC values within 4 to 6 h. This method can be implicated to positive blood culture samples for a direct assay, leading to a reduced time to answer of 6 h assay time [102].

##### 2.3.2.2. Rapid Molecular mRNA-Based AST

We recently reported on a novel and rapid mRNA-based molecular AST approach, determining *Y. pestis* susceptibility towards the recommended therapeutic antibiotics ciprofloxacin and doxycycline [21,24]. The molecular approach is based on qRT-PCR quantitation of early alterations in gene expression of selected mRNA-markers that occur in the bacteria following its exposure to the tested antibiotics. Thus, a prior transcriptomic analysis (RNA-seq.) should be performed to identify the specific mRNA markers for the tested bacteria–antibiotic combination. Opposed to the standard bacterial growth-dependent assays, transcriptional responses to the antibiotic stress occur immediately, within minutes [104] thus antibiotic susceptibility can be determined following a short period of antibiotic exposure, reducing tremendously the time-to-answer. The molecular test is performed according to CLSI guidelines, using standard bacterial inoculum of 5 × 10^5^–1 × 10^6^ cfu/mL and the recommended MH growth medium. Using agar plate or direct blood culture-derived bacterial inoculum, we demonstrated that the molecular AST resulted MIC is correlated to the standard broth microdilution derived MIC [21,24]. A short 7 h assay, including 2 h of growth media adjustment, 2 h exposure to ciprofloxacin or doxycycline, followed by 3 h for RNA extraction and qRT-PCR quantitation, could replace the 20–24 h period required for the growth-dependent standard microdilution assay. Moreover, we demonstrated the applicability of the molecular test on blood culture samples spiked with *Y. pestis*, using a prior plasma separation step as described in Section 2.2.2 and showed its relevance as a rapid test in clinical settings [24]. The growing implementation of rapid mRNA-based automated diagnostics, especially nowadays with the COVID-19 pandemic, places mRNA-based AST as an attractive alternative to the standard ASTs.

##### 2.3.2.3. Optical and Microscopic Screening

Advances in high-resolution optical screening, microscopy, image process, and Artificial Intelligence (AI) offer new options for assessing the growth and morphological characteristic of bacteria that may be used for rapid ASTs. One of these technologies that was implicated in bioterror agents is the oCelloScope. The basic principle behind the oCelloScope detection system (BioSense Solutions ApS, Farum, Denmark) is digital time-lapse microscopy-scanning which captures through a fluid sample a series of images representing the dynamic bacterial growth within the tested wells containing different antibiotics at different concentrations (for detail see [105]). The method was implemented as an AST for *B. anthracis* and provided MIC values within less than 4 h [106]. Although this AST method is relatively rapid, the assay is performed within a liquid culture thus time-consuming preliminary bacterial enrichment and isolation steps are required, leading to an overall time of test of hours to a day. Others have used a laser light scattering technology to monitor antibiotic-dependent growth and reported on MIC values and susceptibility categories within 4 h for *B. anthracis* and 10 h for *Y. pestis* and *Burkholderia pseudomallei* [107]. As for the oCelloScope technology, the laser scattering method requires preliminary enrichment and isolation steps as well, increasing the time to answer.

##### 2.3.2.4. Phage Based ASTs

Phages have been in service for pathogen identifications for decades. Confirmed identification of *Y. pestis* strains is conducted by the CDC and the USAMRIID, by using the lytic phage ɸA1122 for the phage growth-dependent lysis assay [108]. Similarly, the FDA-approved γ phage lysis assay is used by the CDC and the Laboratory Response Network (LRN) as a standard for confirmatory identification of *B. anthracis* [109]. A shorter diagnostic assay, based on a “light-tagged” reporter phage that infects the phage sensitive and metabolic active bacterial cells and then uses the host’s transcriptional and translational machinery to elicit a bioluminescent response, offers quicker diagnostic options [110,111]. The ability of phages to differentiate between live/dead bacteria enabled their usage also as an AST tool. In an antibiotic exposure setting as recommended by the CLSI for conducting standard microdilution, the *Y. pestis* specific reporter-phage ϕA1122::luxAB was added 60 min after the exposure to chloramphenicol or tetracycline and 120 min after exposure to streptomycin [110]. The bioluminescent signal was correlated with the concentration of live *Y. pestis* and at antibiotic concentrations equal to the MIC value or higher, a reduction in the bioluminescent signal to threshold level was noticed [110]. The assay was implemented in a clinical setting of whole blood samples that were spiked with ~100 cfu/mL of *Y. pestis* and diluted 1:20 in LB medium containing increasing concentrations of chloramphenicol, streptomycin, or tetracycline. Antibiotic susceptibility was obtained within 5 h, directly from whole blood sample without the need for any enrichment or isolation step [112]. Moreover, this assay was applicable on blood cultures spiked with *Y. pesits* towards ciprofloxacin, doxycycline, chloramphenicol, streptomycin, and gentamicin [113].

##### 2.3.2.5. Micro-Agar-PCR-Test (MAPt)

The ability to perform an AST from a dynamic range of bacterial concentration holds great advantages as no time-consuming enrichment or quantification steps are needed. Moreover, the ability to directly apply a sample to the AST without isolation from the original sample is very beneficial. We recently reported on the development of a rapid AST that fulfills all these criteria. The new assay, named MAPt (micro-agar-PCR-test), is based on a micro-agar dilution test followed by a sensitive and specific qPCR step that detects only the target bacteria from all other naturally existing bacteria in the sample thus offering a target MIC value even in a heterologous sample [114]. Furthermore, agar medium better supports the growth of low bacterial concentrations which could not be reached by using the broth microdilution-based assays thus allowing MIC determination of samples with low bacterial concentrations. Additionally, MAPt was shown to provide adequate MIC values to all three Tier-1 agents, directly from blood culture samples, with no need for purification steps. Moreover, the assay was even shown to be applicable on whole blood samples with no need for the enrichment step in the blood culture bottles at concentrations as low as 2.5 × 10^2^ cfu/mL. Thus, MAPt may provide in a clinical setting a substantially rapid sample-to-answer platform, with timeframes of clinical relevance [115].

### 2.4. Intracellular ASTs

Of the three Tier-1 agents only *F. tularensis* is a facultative intracellular bacterium. Data regarding the minimal inhibitory extracellular concentration (MIEC) needed to eradicate intracellular bacteria, may add another level of prediction to the efficacy of the suggested antibiotic treatment. Basically, the MIEC value mirrors the dynamic effects of antibiotics on the host cell-intracellular microorganism association, thus, low values of MIC and MIEC may point to a preferable antibiotic. Generally, the intracellular activity of a tested antibiotic against intracellular facultative microorganisms is determined by the viable bacterial counts. As *F. tularensis* is a slow-growing bacterium this assay is time-consuming and may take 2–3 days. To overcome this timewise obstacle we developed a rapid real-time quantitative PCR assay that replaces the traditional viable counts and managed to determine MIEC within 3 h instead of the 2–3 days [30]. Others [116] have adopted the dye uptake assay, which is based on the cytotoxic effect of the bacteria on eukaryotic cells, to monitor the intracellular effect of the tested antibiotic. Although the dye uptake test simplifies the antibiotic screening procedure compared to the traditional bacterial counts, it is still time-consuming and requires a few days.

### 2.5. Avoiding the Latter-ASTs of Environmental Samples

As bioterror via an infectious agent may lead to disastrous consequences, including morbidity, mortality as well as economic, social and psychological apprehensions, preparedness and means to mitigate the event at the earliest stage are advantageous. Proper prophylaxis treatment, offered promptly to potentially infected individuals before symptom occurrence, may be a game-changer. This scenario could be applicable only if an AST can be performed on the infectious agent in an environmental sample. As environmental samples are overloaded with various naturally occurring bacteria, usually prior isolation steps are mandatory to obtain an antibiotic susceptibility profile of the bioterror agent and not the naturally existing bacteria. As suggested for clinical samples, bacterial isolation through selective media is an option, yet, this may take a few days.

To overcome this hurdle, we applied the rapid and sensitive MAPt (Section 2.3.2.5). The assay was applied on various environmental samples, outdoors as well as indoors, spiked with all three Tier-1 agents, with low to high bacterial concentrations, and was found to provide adequate MIC values, such as the ones obtained by the standard AST, albeit at remarkably shorter time frames. Strikingly, this outstanding assay is capable of providing adequate MIC values even when the ratio of the tested bacteria to the naturally occurring environmental bacteria is 1:1 [114]. Similarly, the reporter-phage AST, was also found to be a potential method for antibiotic susceptibility determination of *Y. pestis* inoculated environmental samples [113]. To the best of our knowledge these are the first assays that provide such performances, namely, they work even in the presence of bacterial contaminations, thus there is no need for the time-consuming isolation/purification steps [114]. Moreover, MAPt was shown to be applicable to a wide range of bacterial concentrations. Hence, MAPt and reporter-phage-based AST may serve as preparedness means in case of a bioterror event, a natural emerging infectious disease, or for surveillance strategies, providing decision-making personal treatment regimens before the onset of symptoms in infected individuals. The AST methods mentioned above are summarized in Table 1.

## 3. Concluding Remarks

The possibility of a bioterror event involving an antibiotic-resistant bacterium is of great concern. The utmost preparedness goal for this scenario would be the availability of a rapid AST that would provide an antibiotic susceptibility profile within a time frame of clinical relevance. Although an ample number of rapid ASTs are described in the literature, most of them are not applicable directly to clinical samples, let alone to environmental ones, and do require additional isolation/purification/enrichment steps, which are time-consuming and hold major drawbacks, timewise. These constraints become even more augmented under a bioterror event potentially involving an engineered antibiotic resistance bacterium, as public health decisions should promptly be taken. ASTs that do not have an inoculum size effect, can directly be applied to clinical and environmental samples and can simultaneously test multiple specimens, antibiotics, and most importantly, samples harboring mixed bacteria should be favored.

## Figures and Tables

**Table 1:** ASTs implemented on Tier-1 bioterror bacterial agents.

Available ASTs for Bioterror Bacterial Agents (*B. anthracis*, *Y. pestis*, and *F. tularensis)*		Process Features					Sample Type		
		Isolation/Enrichment Steps	Bacteria Concentration Dependence	Preceding Preparation Time	AST Time	Total Time (h)	Bacterial Culture	Clinical (Blood, Blood Culture)	Environmental
**Genotypic**	High-throughput sequencing [84,85]	DNA extraction	Yes (min. 1 ng DNA)	1 h	10–16 h	11–17 h *	+	+ Blood, blood culture	+
**Phenotypic**	Broth medium dilution [37]	Blood culture enrichment/isolation from environment	Yes	24–48 h	24–48 h	48–96 h	+	+	+
	Molecular mRNA based [21,24]	Blood culture enrichment/isolation from environment	Yes	18 h	*Y. pestis*- 7 h	~25 h	+	+ Blood culture	N.D
	Live/Dead fluorescent detection (SIR) [103]	Yet to be determined	No (Minimal 5 × 10^4^)	Yet to be determined	*Y. pestis*- 7 h	Yet to be determined	+	N.D	N.D
	Optical and microscopic screening [106]	Yet to be determined	Yes	Yet to be determined	*B.* *anthracis* 4 h	Yet to be determined	+	N.D	N.D
	Reporter-phage [111,112,113]	No need	No (Minimal 10^2^ cfu/mL)	No need	*B.* *anthracis* 80–160 min *Y. pestis* 5–16 h	*B. anthracis* 80–160 min *Y. pestis* 5–16 h	+	+ Blood, blood culture	+
	MAPt [114,115]	No need	No (Minimal 5 × 10^2^ cfu/mL)	No need	*B. anthracis* −7 h, *Y. pestis* 13 h, *F. tularemia* 17 h	*B. anthracis* −7 h, *Y. pestis* 13 h, *F. tularemia* 17 h	+	+ Blood, blood culture	+

* Including bioinformatic analysis, dependent on database size and availability. + Tested and published.

## Data Availability

Not applicable.

## List of Abbreviations

AI	Artificial Intelligence
AST	Antibiotic susceptibility test
BMD	Broth microdilution
caMHB	cation-adjusted Mueller–Hinton broth
CDC	Centers of Disease control and presentation
CHA	Cysteine Heart Agar
CHAB	CHA with 9% sheep blood
CHAB	A-CHAB with ampicillin
CIN	Cefsulodin-irgasan-novobiocin
CFU	Colony-forming unit
CLSI	Clinical and Laboratory Standards Institute
EUCAST	European Committee on Antimicrobial Susceptibility Testing
FACS	Fluorescent Activated Cell Sorter
FRET	Fluorescence Resonance Energy Transfer
HTS	High-throughput Sequencing
IMS	Immunomagnetic Separation
LB	Luria-Bertani
LRN	Laboratory Response Network
MAPt	Micro-Agar-PCR Test
MH	Mueller–Hinton
MIC	Minimal Inhibitory Concentration
MIEC	Minimal Inhibitory Extracellular Concentration
PO	*per os*
RAST	Rapid Antimicrobial Susceptibility Testing
RNA	seq-RNA sequencing
SIR	Spectral Intensity Ratio
WHO	World Health Organization

## References

[1] Clemente-Suárez V.J., Dalamitros A.A., Beltran-Velasco A.I., Mielgo-Ayuso J., Tornero-Aguilera J.F. (2020). Social and Psychophysiological Consequences of the COVID-19 Pandemic: An Extensive Literature Review. Front. Psychol..

[2] Aloni-Grinstein R., Rotem S. (2021). COVID-19 Pandemic: A Lesson for Antibiotic and Antiseptic Stewardship. Am. J. Public Health Res..

[3] Trevisanato S.I. (2007). The ‘Hittite plague’, an epidemic of tularemia and the first record of biological warfare. Med. Hypotheses.

[4] Barras V., Greub G. (2014). History of biological warfare and bioterrorism. Clin. Microbiol. Infect..

[5] CDC Bioterrorism Agents/Diseases. https://emergency.cdc.gov/agent/agentlist-category.asp.

[6] National Academies of Sciences, Engineering, and Medicine (2018). Biodefense in the Age of Synthetic Biology.

[7] Dixon T.C. (1999). Anthrax. N. Engl. J. Med..

[8] Holty J.E.C., Bravata D.M., Liu H., Olshen R.A., McDonald K.M., Owens D.K. (2006). Systemic review: A century of inhalational anthrax cases from 1900 to 2005. Ann. Intern. Med..

[9] Darling R.G., Catlett C.L., Huebner K.D., Jarrett D.G. (2002). Threats in bioterrorism I: CDC category A agents. Emerg. Med. Clin. North Am..

[10] Goal A.K. (2015). Anthrax: A disease of biowarfare and public health importance. World J. Clin. Cases.

[11] Respicio-Kingry L.B., Yockey B.M., Acayo S., Kaggwa J., Apangu T., Kugeler K.J., Eisen R.J., Griffith K.S., Mead P.S., Schriefer M.E. (2016). Two distinct Yersinia pestis populations causing plague among humans in the West Nile region of Uganda. PLoS Negl. Trop. Dis..

[12] Andrianaivoarimanana V., Piola P., Wagner D.M., Rakotomanana F., Maheriniaina V., Andrianalimanana S., Chanteau S., Rahalison L., Ratsitorahina M., Rajerison M. (2019). Trends in human plague, Madagascar, 1998–2016. Emerg. Infect. Dis..

[13] Shi L., Yang G., Zhang Z., Xia L., Liang Y., Tan H., He J., Xu J., Song Z., Li W. (2018). Reemergence of human plague in Yunnan, China in. PLoS ONE.

[14] Inglesby T.V., Dennis D.T., Henderson D.A., Bartlett J.G., Ascher M.S., Eitzen E., Fine A.D., Friedlander A.M., Hauer J., Koerner J.F. (2000). Plague as a Biological Weapon. JAMA.

[15] WHO (1970). Health Aspects of Chemical and Biological Weapons.

[16] CDC CDC, Plague. https://www.cdc.gov/plague/index.html.

[17] Sebbane F., Lemaître N. (2021). Antibiotic Therapy of Plague: A Review. Biomolecules.

[18] Guiyoule A. (2001). Transferable Plasmid-Mediated Resistance to Streptomycin in Clinical Isolate of Yersinia pestis. Emerg. Infect. Dis..

[19] Galimand M., Carniel E., Courvalin P. (2006). Resistance of Yersinia pestis to Antimicrobial Agents. Antimicrob. Agents Chemother..

[20] Lindler L.E., Fan W., Jahan N. (2001). Detection of Ciprofloxacin-Resistant Yersinia pestis by Fluorogenic PCR Using the LightCycler. J. Clin. Microbiol..

[21] Steinberger-Levy I., Shifman O., Zvi A., Ariel N., Beth-Din A., Israeli O., Gur D., Aftalion M., Maoz S., Ber R. (2016). A rapid molecular test for deternining Yersinia pestis susceptibility to ciprofloxacin by the quantification of differntially expressed marker genes. Front. Microbiol..

[22] Louie A., Heine H.S., VanScoy B., Eichas A., Files K., Fikes S., Brown D.L., Liu W., Kinzig-Schippers M., Sorgel F. (2011). Use of an in vitro pharmacodynamic model to derive a moxifloxacin regimen that optimizes kill of Yersinia pestis and prevents emergence of resistance. Antimicrob. Agents Chemother..

[23] Louie A., Deziel M.R., Liu W., Drusano G.L. (2007). Impact of Resistance Selection and Mutant Growth Fitness on the Relative Efficacies of Streptomycin and Levofloxacin for Plague Therapy. Antimicrob. Agents Chemother..

[24] Shifman O., Steinberger-Levy I., Aloni-Grinstein R., Gur D., Aftalion M., Ron I., Mamroud E., Ber R., Rotem S. (2019). A Rapid Antimicrobial Susceptibility Test for Determining Yersinia pestis Susceptibility to Doxycycline by RT-PCR Quantification of RNA Markers. Front. Microbiol..

[25] McLendon M.K., Apicella M.A., Allen L.A. (2006). Francisella tularensis: Taxonomy, genetics, and Immunopathogenesis of a po-tential agent of biowarfare. Annu. Rev. Microbiol..

[26] Maurin M. (2015). Francisella tularensis as a potential agent of bioterrorism?. Expert Rev. Anti-Infect. Ther..

[27] Egan J.R., Hall I.M., Leach S. (2011). Modeling Inhalational Tularemia: Deliberate Release and Public Health Response. Biosecurity Bioterrorism Biodefense Strat. Pr. Sci..

[28] WHO (2007). WHO Guidelines on Tularaemia. Epidemic and Pandemic Alert and Response.

[29] Fàbrega A., Madurga S., Giralt E., Vila J. (2009). Mechanism of action of and resistance to quinolones. Microb. Biotechnol..

[30] Aloni-Grinstein R., Shifman O., Lazar S., Steinberger-Levy I., Maoz S., Ber R. (2015). A rapid real-time quantitative PCR assay to determine the minimal inhibitory extracellular concentration of antibiotics against an intracellular Francisella tularensis Live Vaccine Strain. Front. Microbiol..

[31] Kassinger S.J., van Hoek M.L. (2021). Genetic Determinants of Antibiotic Resistance in Francisella. Front. Microbiol..

[32] Loveless B.M., Yermakova A., Christensen D.R., Kondig J.P., Heine H.S., Wasieloski L.P., Kulesh D.A. (2010). Identification of ciprofloxacin resistance by SimpleProbe™, High Resolution Melt and Pyrosequencing™ nucleic acid analysis in biothreat agents: Bacillus anthracis, Yersinia pestis and Francisella tularensis. Mol. Cell. Probes.

[33] Sutera V., Levert M., Burmeister W., Schneider D., Maurin M. (2014). Evolution toward high-level fluoroquinolone resistance in Francisella species. J. Antimicrob. Chemother..

[34] Gestin B., Valade E., Thibault F., Schneider D., Maurin M. (2010). Phenotypic and genetic characterization of macrolide resistance in Francisella tularensis subsp. holarctica biovar I. J. Antimicrob. Chemother..

[35] Sutera V., Hennebique A., Lopez F., Fernandez N., Schneider D., Maurin M. (2020). Genomic trajectories to fluoroquinolone resistance in Francisella tularensis subsp. holarctica live vaccine strain. Int. J. Antimicrob. Agents.

[36] Biot F.V., Bachert B.A., Mlynek K.D., Toothman R.G., Koroleva G.I., Lovett S.P., Klimko C.P., Palacios G.F., Cote C.K., Ladner J.T. (2020). Evolution of Antibiotic Resistance in Surrogates of Francisella tularensis (LVS and *Francisella novicida*): Effects on Biofilm Formation and Fitness. Front. Microbiol..

[37] CLSI (2015). Methods for Antimicrobial Dilution and Disk Susceptibility Testing of Infrequently Isolated or Fastidious Bacteria.

[38] Mohammed M.J., Marston C.K., Popovic T., Weyant R.S., Tenover F.C. (2002). Antimicrobial Susceptibility Testing of Bacillus anthracis: Comparison of Results Obtained by Using the National Committee for Clinical Laboratory Standards Broth Microdilution Reference and Etest Agar Gradient Diffusion Methods. J. Clin. Microbiol..

[39] Scheel O., Hoel T., Sandvik T., Berdal B.P. (1993). Susceptibility pattern of Scandinavian Francisella tularensis isolates with regard to oral and parenteral antimi-crobial agents. APMIS.

[40] Ikaheimo I., Syrjälä H., Karhukorpi J., Schildt R., Koskela M. (2000). In vitro antibiotic susceptibility of *Francisella tularensis* isolated from humans and animals. J. Antimicrob. Chemother..

[41] Johansson A.F., Urich S.K., Chu M.C., Sjöstedt A., Tärnvik A. (2002). In Vitro Susceptibility to Quinolones of *Francisella tularensis* subspecies tularensis. Scand. J. Infect. Dis..

[42] Tomaso H., Al Dahouk S., Hofer E., Splettstoesser W.D., Treu T.M., Dierich M.P., Neubauer H. (2005). Antimicrobial susceptibilities of Austrian Francisella tularensis holarctica biovar II strains. Int. J. Antimicrob. Agents.

[43] Del Blanco N.G. (2004). In vitro susceptibility of field isolates of *Francisella tularensis* subsp. holarctica recovered in Spain to several antimicrobial agents. Res. Veter. Sci..

[44] Urich S.K., Petersen J.M. (2008). In Vitro Susceptibility of Isolates of *Francisella tularensis* Types A and B from North America. Antimicrob. Agents Chemother..

[45] Valade E., Vaissaire J., Mérens A., Hernandez E., Gros C., Le Doujet C., Paucod J.-C., Thibault F.M., Durand B., Lapalus M. (2008). Susceptibility of 71 French isolates of *Francisella tularensis* subsp. holarctica to eight antibiotics and accuracy of the Etest^®^ method. J. Antimicrob. Chemother..

[46] Velinov T., Nicolova M., Kuzmanov A. (2011). In vitro antimicrobioal susceptibility of *Francisella tularensis* isolated in Bulgaria. Probl. Inf. Parasit. Dis..

[47] Yeşilyurt M., Kilic S., Çelebi B., Çelik M., Gül S., Erdoğan F., Özel G., Kılıç S. (2011). Antimicrobial susceptibilities of *Francisella tularensis* subsp. holarctica strains isolated from humans in the Central Anatolia region of Turkey. J. Antimicrob. Chemother..

[48] Georgi E., Schacht E., Scholz H.C., Splettstoesser W.D. (2012). Standardized broth microdilution antimicrobial susceptibility testing of *Francisella tularensis* subsp. holarctica strains from Europe and rare Francisella species. J. Antimicrob. Chemother..

[49] Hotta A., Fujita O., Uda A., Sharma N., Tanabayashi K., Yamamoto Y., Yamada A., Morikawa S. (2013). In vitro antibiotic susceptibility of Francisella tularensis isolates from Japan. Jpn. J. Infect. Dis..

[50] Kilic S., Celebi B., Acar B., Ataş M. (2012). In vitro susceptibility of isolates of Francisella tularensis from Turkey. Scand. J. Infect. Dis..

[51] Origgi F.C., Pilo P., Origgi F.C., Pilo P. (2016). *Francisella Tularensis* Clades B.FTN002-00 and B.13 Are Associated with Distinct Pathology in the European Brown Hare (*Lepus europaeus*). Veter. Pathol..

[52] Kreizinger Z., Makrai L., Helyes G., Magyar T., Erdélyi K., Gyuranecz M. (2012). Antimicrobial susceptibility of *Francisella tularensis* subsp. holarctica strains from Hungary, Central Europe. J. Antimicrob. Chemother..

[53] Caspar Y., Maurin M. (2017). *Francisella tularensis* Susceptibility to Antibiotics: A Comprehensive Review of the Data Obtained In vitro and in Animal Models. Front. Cell. Infect. Microbiol..

[54] Jonasson E., Matuschek E., Kahlmeter G. (2020). The EUCAST rapid disc diffusion method for antimicrobial susceptibility testing directly from positive blood culture bottles. J. Antimicrob. Chemother..

[55] Shifman O., Aminov T., Aftalion M., Gur D., Cohen H., Bar-David E., Cohen O., Mamroud E., Levy H., Aloni-Grinstein R. (2021). Evaluation of the European Committee on Antimicrobial Susceptibility Testing Guidelines for Rapid Antimi-crobial Susceptibility Testing of Bacillus anthracis-, Yersinia pestis- and *Francisella tularensis*-Positive Blood Cultures. Microor. Ganisms..

[56] Pearce T.W., Powell E.O. (1951). A Selective Medium for *Bacillus anthracis*. J. Gen. Microbiol..

[57] Knisely R.F. (1966). A Selective Medium for *Bacillus Anthracis*. Sel. Medium Bacillus Anthracis.

[58] Zasada A.A. (2020). Detection and Identification of *Bacillus anthracis*: From Conventional to Molecular Microbiology Methods. Microorganism.

[59] Tomaso H., Bartling C., Al Dahouk S., Hagen R.M., Scholz H.C., Beyer W., Neubauer H. (2006). Growth characteristics of *Bacillus anthracis* compared to other Bacillus spp. on the selective nutrient media Anthrax Blood Agar^®^ and Cereus Ident Agar^®^. Syst. Appl. Microbiol..

[60] Klee S., Nattermann H., Becker S., Urban-Schriefer M., Franz T., Jacob D., Appel B. (2006). Evaluation of different methods to discriminate *Bacillus anthracis* from other bacteria of the Bacillus cereus group. J. Appl. Microbiol..

[61] Dragon D., Rennie R. (2001). Evaluation of spore extraction and purification methods for selective recovery of viable Bacillus anthracis spores. Lett. Appl. Microbiol..

[62] Rohde A., Papp S., Feige P., Grunow R., Kaspari O. (2020). Development of a novel selective agar for the isolation and detection of Bacillus anthracis. J. Appl. Microbiol..

[63] Meyer K.F., Batchelder A.P. (1926). Selective Mediums in the Diagnosis of Rodent Plague: Plague Studies. J. Infect. Dis..

[64] Markenson J., Ben-Efraim S. (1963). Oxgall Medium for Identification of Pasteurella Pestis. J. Bacteriol..

[65] Morris E.J. (1958). Selective Media for some Pasteurella Species. J. Gen. Microbiol..

[66] Ber R., Mamroud E., Aftalion M., Tidhar A., Gur D., Flashner Y., Cohen S. (2003). Development of an Improved Selective Agar Medium for Isolation of Yersinia pestis. Appl. Environ. Microbiol..

[67] Schiemann D.A. (1979). Synthesis of a selective agar medium for Yersinia enterocolitica. Can. J. Microbiol..

[68] Aleksic S., Bockemuhl J., Murray P.R., Baron E.J., Pfaller M.A., Tenover F.C., Yolken R.H. (1999). Yersinia and other Enterobacteriaceae. Manual of Clinical Microbiology.

[69] Aftalion M., Aloni-Grinstein R., Andrianaivoarimanana V., Iharisoa A.L., Shmaya S., Gur D., Laskar O., Rajerison M., Mamroud E. (2021). Improved selective BIN agar for a better rate of Yersinia pestis isolation from primary clinical specimens in suspected Madagascar’s plague cases. J. Clin. Microbiol..

[70] Suna G. (1996). *Francisella tularensis* isolation from various clinical specimens. Clin. Microbiol. Infert..

[71] Petersen J.M., Schriefer M.E., Gage K.L., Montenieri J.A., Carter L.G., Stanley M., Chu M.C. (2004). Methods for Enhanced Culture Recovery of *Francisella tularensis*. Appl. Environ. Microbiol..

[72] Petersen J.M., Carlson J., Yockey B., Pillai S., Kuske C., Garbalena G., Pottumarthy S., Chalcraft L. (2009). Direct isolation of Francisella spp. from environmental samples. Lett. Appl. Microbiol..

[73] Humrighouse B.W., Adcock N.J., Rice E.W. (2011). Use of Acid Treatment and a Selective Medium to Enhance the Recovery of *Francisella tularensis* from Water. Appl. Environ. Microbiol..

[74] Aloni-Grinstein R., Schuster O., Yitzhaki S., Aftalion M., Maoz S., Steinberger-Levy I., Ber R. (2017). Isolation of *Francisella tularensis* and Yersinia pestis from Blood Cultures by Plasma Purification and Immunomagnetic Separation Accelerates Antibiotic Susceptibility Determination. Front. Microbiol..

[75] Ber R., Aftalion M., Cohen S., Flashner Y., Mamroud E., Gur D., Steinberger-Levy I., Zahavy E. (2007). Enrichment of Yersinia pestis from Blood Cultures Enables Rapid Antimicrobial Susceptibility Determination by Flow Cytometry. Chem. Biol. Pteridines Folates.

[76] Zahavy E., Fisher M., Bromberg A., Olshevsky U. (2003). Detection of Frequency Resonance Energy Transfer Pair on Double-Labeled Microsphere and Bacillus anthracis Spores by Flow Cytometry. Appl. Environ. Microbiol..

[77] Zahavy E., Heleg-Shabtai V., Zafrani Y., Marciano D., Yitzhaki S. (2009). Application of Fluorescent Nanocrystals (q-dots) for the Detection of Pathogenic Bacteria by Flow-Cytometry. J. Fluoresc..

[78] Zahavy E., Ber R., Gur D., Abramovich H., Freeman E., Maoz S., Yitzhaki S. (2011). Application of Nanoparticles for the Detection and Sorting of Pathogenic Bacteria by Flow-Cytometry. Adv. Exp. Med. Biol..

[79] Peruski L.F., Peruski A.H. (2003). Rapid diagnostic assays in the genomic biology era: Detection and identification of infectious disease and biological weapon agents. Biotechnology.

[80] Seiner D.R., Colburn H.A., Baird C., Bartholomew R.A., Straub T., Victry K., Hutchison J.R., Valentine N., Bruckner-Lea C.J. (2013). Evaluation of the FilmArray(R) system for detection of *Bacillus anthracis*, *Francisella tularensis* and *Yersinia pestis*. J. Appl. Microbiol..

[81] Kozińska A., Seweryn P., Sitkiewicz I. (2019). A crash course in sequencing for a microbiologist. J. Appl. Genet..

[82] Buermans H.P., den Dunnen J.T. (2014). Next generation sequencing technology: Advances and applications. Biochim. Biophys Acta.

[83] Van Dijk E.L., Auger H., Jaszczyszyn Y., Thermes C. (2014). Ten years of next-generation sequencing technology. Trends Genet..

[84] Israeli O., Makdasi E., Cohen-Gihon I., Zvi A., Lazar S., Shifman O., Levy H., Gur D., Laskar O., Beth-Din A. (2020). A rapid high-throughput sequencing-based approach for the identification of unknown bacterial pathogens in whole blood. Futur. Sci. OA.

[85] Israeli O., Cohen-Gihon I., Zvi A., Lazar S., Shifman O., Levy H., Tidhar A., Beth-Din A. (2019). Rapid identification of unknown pathogens in environmental samples using a high-throughput sequencing-based approach. Heliyon.

[86] Didelot X., Bowden R., Wilson D., Peto T.E.A., Crook D.W. (2012). Transforming clinical microbiology with bacterial genome sequencing. Nat. Rev. Genet..

[87] Fricke W.F., Rasko D.A. (2014). Bacterial genome sequencing in the clinic: Bioinformatic challenges and solutions. Nat. Rev. Genet..

[88] Hendriksen R.S., Bortolaia V., Tate H., Tyson G.H., Aarestrup F., McDermott P.F. (2019). Using Genomics to Track Global Antimicrobial Resistance. Front. Public Health.

[89] Matamoros S., Hendriksen R.S., Pataki B.A., Pakseresht N., Rossello M., Silvester N., Amid C., Aarestrup F.M., Koopmans M., Cochrane G. (2020). Accelerating surveillance and research of antimicrobial resistance—An online repository for sharing of anti-microbial susceptibility data associated with whole-genome sequences. Microb. Genom..

[90] McArthur A., Tsang K.K. (2016). Antimicrobial resistance surveillance in the genomic age. Ann. N. Y. Acad. Sci..

[91] Bortolaia V., Kaas R.S., Ruppe E., Roberts M.C., Schwarz S., Cattoir V., Philippon A., Allesoe R.L., Rebelo A.R., Florensa A.F. (2020). ResFinder 4.0 for predictions of phenotypes from genotypes. J. Antimicrob. Chemother..

[92] Kim J., Greenberg D.E., Pifer R., Jiang S., Xiao G., Shelburne S.A., Koh A., Xie Y., Zhan X. (2020). VAMPr: VAriant Mapping and Prediction of antibiotic resistance via explainable features and machine learning. PLoS Comput. Biol..

[93] Hunt M., Mather A.E., Sánchez-Busó L., Page A.J., Parkhill J., Keane J.A., Harris S.R. (2017). ARIBA: Rapid antimicrobial resistance genotyping directly from sequencing reads. Microb. Genom..

[94] Feldgarden M., Brover V., Haft D.H., Prasad A.B., Slotta D.J., Tolstoy I., Tyson G.H., Zhao S., Hsu C., McDermott P.F. (2019). Validating the AMRFinder Tool and Resistance Gene Database by Using Antimicrobial Resistance Geno-type-Phenotype Correlations in a Collection of Isolates. Antimicrob. Agents Chemother..

[95] Doster E., Lakin S.M., Dean C.J., Wolfe C., Young J.G., Boucher C., Belk K.E., Noyes N.R., Morley P.S. (2020). MEGARes 2.0: A database for classification of antimicrobial drug, biocide and metal resistance determinants in metagenomic sequence data. Nucleic Acids Res..

[96] Gargis A.S., Cherney B., Conley A.B., McLaughlin H.P., Sue D. (2019). Rapid Detection of Genetic Engineering, Structural Variation, and Antimicrobial Resistance Markers in Bacterial Biothreat Pathogens by Nanopore Sequencing. Sci. Rep..

[97] Chen Y., Succi J., Tenover F.C., Koehler T.M. (2003). Beta-lactamase genes of the penicillin-susceptible *Bacillus anthracis* Sterne strain. J. Bacteriol..

[98] Nuding S., Zabel L.T. (2013). Detection, identification, and susceptibility testing of bacteria by flow cytometry. J. Bacteriol. Parasitol..

[99] Müller S., Nebe-Von-Caron G. (2010). Functional single-cell analyses: Flow cytometry and cell sorting of microbial populations and communities. FEMS Microbiol. Rev..

[100] Nebe-Von-Caron G., Stephens P., Hewitt C., Powell J., Badley R. (2000). Analysis of bacterial function by multi-colour fluorescence flow cytometry and single cell sorting. J. Microbiol. Methods.

[101] Eran Z. (2019). Spectral Intensity Ratio (SIR) Analysis for Rapid Live Microbial Enumeration. U.S. Patent.

[102] Ingber G., Ben-David M., Fridman M., Gluckman Y., Gohman D., Munz O.H., Shinderman A., Zahavy E. (2021). Rapid Antimicrobial Susceptibility Testing based on A Unique Spectral Intensity Ratio Analysis via Single Fluorescence Membrane Dye Staining and Flow Cytometry.

[103] Zahavy E., Rotem S., Gur D., Aloni-Grinstein R., Aftalion M., Ber R. (2018). Rapid Antibiotic Susceptibility Determination for *Yersinia pestis* Using Flow Cytometry Spectral Intensity Ratio (SIR) Fluorescence Analysis. J. Fluoresc..

[104] Khazaei T., Barlow J., Schoepp N., Ismagilov R.F. (2018). RNA markers enable phenotypic test of antibiotic susceptibility in Neisseria gonorrhoeae after 10 minutes of ciprofloxacin exposure. Sci. Rep..

[105] Fredborg M., Andersen K.R., Jørgensen E., Droce A., Olesen T., Jensen B.B., Rosenvinge F.S., Sondergaard T.E. (2013). Real-Time Optical Antimicrobial Susceptibility Testing. J. Clin. Microbiol..

[106] McLaughlin H.P., Gargis A., Michel P., Sue D., Weigel L.M. (2017). Optical Screening for Rapid Antimicrobial Susceptibility Testing and for Observation of Phenotypic Diversity among Strains of the Genetically Clonal Species Bacillus anthracis. J. Clin. Microbiol..

[107] Bugrysheva J.V., Lascols C., Sue D., Weigel L.M. (2016). Rapid Antimicrobial Susceptibility Testing of *Bacillus anthracis*, *Yersinia pestis*, and *Burkholderia pseu-domallei* by Use of Laser Light Scattering Technology. J. Clin. Microbiol..

[108] Chu M.C. (2000). Laboratory Manual Pf Plague Diagnostic Tests.

[109] Abshire T.G., Brown J.E., Ezzell J.W., Mandal P., Banerjee U., Casadevall A., Nosanchuk J.D. (2005). Production and Validation of the Use of Gamma Phage for Identification of *Bacillus anthracis*. J. Clin. Microbiol..

[110] Schofield D.A., Molineux I.J., Westwater C. (2012). Rapid identification and antibiotic susceptibility testing of Yersinia pestis using bioluminescent reporter phage. J. Microbiol. Methods.

[111] Schofield D.A., Sharp N.J., Vandamm J., Molineux I.J., Spreng K.A., Rajanna C., Westwater C., Stewart G.C. (2013). Bacillus anthracis diagnostic detection and rapid antibiotic susceptibility determination using ’biolumines-cent’ reporter phage. J. Microbiol. Methods.

[112] Vandamm J.P., Rajanna C., Sharp N.J., Molineux I.J., Schofield D.A. (2014). Rapid Detection and Simultaneous Antibiotic Susceptibility Analysis of *Yersinia pestis* Directly from Clinical Specimens by Use of Reporter Phage. J. Clin. Microbiol..

[113] Moses S., Aftalion M., Mamroud E., Rotem S., Steinberger-Levy I. (2021). Reporter-Phage-Based Detection and Antibiotic Susceptibility Testing of *Yersinia pestis* for a Rapid Plague Outbreak Response. Microorganism.

[114] Aloni-Grinstein R., Shifman O., Gur D., Aftalion M., Rotem S. (2020). MAPt: A Rapid Antibiotic Susceptibility Testing for Bacteria in Environmental Samples as a Means for Bioterror Preparedness. Front. Microbiol..

[115] Rotem S., Shifman O., Aftalion M., Gur D., Aminov T., Aloni-Grinstein R. (2021). Rapid antibiotic susceptibility testing of Tier-1 agents *Bacillus anthracis*, *Yersinia pestis*, and *Francisella tularensis* directly from whole blood samples. Front. Microbiol..

[116] Sutera V., Caspar Y., Boisset S., Maurin M. (2014). A new dye uptake assay to test the activity of antibiotics against intracellular *Francisella tularensis*. Front. Cell. Infect. Microbiol..

